# Sexual and Urinary Health among Women following Bariatric Surgery

**DOI:** 10.1007/s11695-024-07226-0

**Published:** 2024-08-19

**Authors:** Alejandro D. Lopez, Jonathan Carter, Rachel Rubin, I. Elaine Allen, Nathan M. Shaw, Lindsay A. Hampson

**Affiliations:** 1 Department of Urology, School of Medicine, University of California San Francisco, 400 Parnassus Ave, Box 0738, San Francisco, CA 94143 USA; 2 Department of General Surgery, School of Medicine, University of California San Francisco, 400 Parnassus Ave, San Francisco, CA 94143 USA; 3 Department of Urology, MedStar Georgetown Department of Urology, 3800 Reservoir Rd. NW, Washington, DC 20007 USA; 4 Department of Epidemiology & Biostatistics, School of Medicine, University of California San Francisco, 400 Parnassus Ave, San Francisco, CA 94143 USA; 5 Department of Reconstructive and Plastic Surgery, MedStar Georgetown Department of Urology, 3800 Reservoir Rd. NW, Washington, DC 20007 USA; 6 Department of Surgery, San Francisco Veterans Affairs Medical Center, 4150 Clement St, San Francisco, CA 94121 USA

**Keywords:** Metabolic and bariatric surgery, Urinary health, Sexual health

## Abstract

**Purpose:**

Women with obesity are more likely to experience bothersome urinary and sexual symptoms, but the long-term effect of metabolic and bariatric surgery (MBS) on these outcomes is poorly understood. We aimed to describe how MBS longitudinally impacted women’s urinary and sexual health.

**Methods:**

Patients who underwent MBS at the University of California, San Francisco Medical Center (UCSF) between 2009 and 2021 participated in a survey examining sexual health, pelvic organ prolapse (POP), and urinary health using three validated questionnaires: a modified version of the Female Sexual Function Index (FSFI), the Pelvic Organ Prolapse Distress Inventory 6 (POPDI-6), and the Urinary Distress Inventory 6 (UDI-6). All questions asked referenced two time points: before surgery and at the time of survey. Logistic regression identified predictors of symptom improvement.

**Results:**

Of 162 participants contacted, 118 (73%) had complete survey data. Mean body mass index (BMI) decreased from 52.4 ± 12.6 to 36.3 ± 9.7 kg/m^2^ (*p* < 0.01) with an average follow-up of 6 years. The mean UDI-6 score amongst women was 24 ± 24 prior to MBS and 24 ± 26 at the time of survey administration (*p* = 0.458). Mean modified FSFI scores amongst women were 15 ± 5 prior to surgery and 14 ± 7 at the time of survey administration (*p* = 0.005). The overall mean POPDI-6 score amongst women was 13 ± 15 prior to surgery and 9 ± 14 at the time of survey administration (*p* = 0.056).

**Conclusion:**

Women who underwent MBS reported a high rate of sexual and urological dysfunction that did not improve longitudinally, despite significant weight loss.

**Graphical Abstract:**

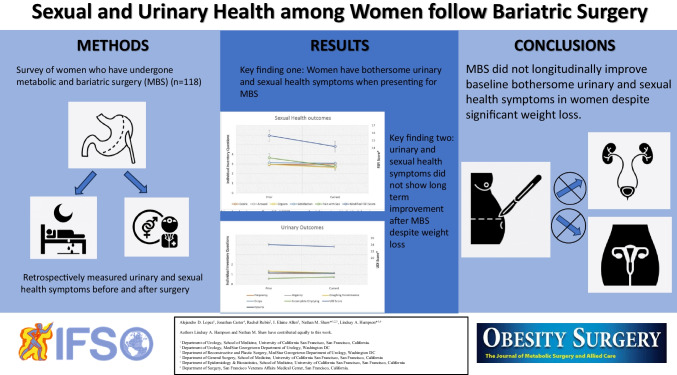

**Supplementary Information:**

The online version contains supplementary material available at 10.1007/s11695-024-07226-0.

## Introduction

The Centers for Disease Control and Prevention estimates that the prevalence of obesity in the USA was 42% between 2017 and 2020, with rates of severe obesity, defined as a body mass index > 40 kg/m^2^, rising from 5 to 9% in the same period [1]. Men and women with severe obesity experience serious medical, physical, and emotional side-effects. Metabolic and bariatric surgery (MBS) is an increasingly common and effective treatment option for severe obesity, with 256,000 operations being performed in 2019 alone [2].

Significant urinary and sexual dysfunction results from the mechanical, anatomic, and infectious changes that arise from obesity [3–5]. In men, outcomes of acquired buried penis syndrome following MBS have been described [6–8]. For women, however, few reports exist on urinary and sexual outcomes before and after MBS[9]. Prior studies on women have focused mostly on psychological and specific sexual outcomes after MBS—these studies have reported that depression, self-image, sexual desire, and satisfaction all improve up to 5 years after MBS. Few studies, however, have investigated broad urinary, sexual, and prolapse outcomes of women following MBS.

The primary aim of this study was to understand how MBS-induced weight loss affected urinary, sexual, and pelvic organ prolapse (POP) outcomes in women. Secondarily, patient factors were used to generate predictive models of who derived the most post-operative benefit from MBS across measured domains. We hypothesized that improvements in all three domains would be seen following MBS, that these improvements would be associated with sustained weight loss, and that premenopausal women would derive the most benefit in sexual health domains.

## Methods

### Population and Recruitment

Patients who underwent MBS at UCSF between 2009 and 2021 were contacted for participation. All contact of patients and recruitment in addition to informed consent was approved by an institutional IRB, reference number: 22–36,207. Patients were considered eligible if they were English or Spanish-speaking, aged 18 years or older, and underwent primary MBS with laparoscopic gastric bypass, laparoscopic sleeve gastrectomy, or LapBand placement. Additionally, patients receiving bariatric care at our center were included if they underwent bariatric esophagogastroduodenoscopy or any bariatric surgical revision in the same time period, even if primary operation was performed previously by another center. Initial contact was via a mailed and emailed letter. Secondary contact was then made by research team via email and phone outreach. Afterwards, all eligible participants were contacted via phone call and, if interested, were sent the study information and online REDCap survey (Appendix 1) by email. If not reached by phone over the initial recruitment period, patients were sent a final email invitation to participate. Patients who proceeded with the survey were asked to retrospectively evaluate symptoms prior to surgery, at their perceived best after surgery and at the current timepoint. As patients were at variable time points following surgery the “current” time points were not necessarily uniform. Objective measurements of weight loss, post operative weights, type of surgery, etc. were obtained from chart review as described in detail below.

As an incentive, participants who completed the survey were entered into a raffle to win one of five $100 Amazon gift cards. This manuscript focuses on analysis of women only.

### Measures

We obtained the following demographic and clinical characteristics via chart review, clinical database review, and survey: current age, age at time of surgery, surgery type, height at time of survey, pre-operative weight, weight at time of survey, race/ethnicity, use of hormonal supplementation, and menstrual status.

Our survey utilized questions from three validated measures to evaluate sexual health, POP, and urinary health: (1) the Female Sexual Function Index (FSFI), whose score ranges from 0 to 25, with 0 being worst and 25 best, (2) the Pelvic Organ Prolapse Distress Inventory 6 (POPDI-6), whose score ranges from 0 to 100 with 0 being best and 100 being worst, and (3) the Urinary Distress Inventory 6 (UDI-6), whose score ranges from 0 to 100 with 0 being best and 100 being worst. The UDI-6 and POPDI-6 inventories were used in their entirety. Five questions were chosen from the FSFI to evaluate five of the six domains normally included in this inventory: desire, arousal, orgasm, satisfaction, and pain. All questions in each inventory were asked in reference to two different time points: before surgery (prior) and at the time of the survey (currently).

### Analysis

All data were summarized overall and by (1) pre-surgery and (2) current (time of survey completion) time points using means and standard deviations for continuous variables and frequencies and percentages for categorical variables. Analysis of variance was used to compare demographics and clinical variables by weight and BMI as well as scores (FSFI, POPDI-6, and UDI-6) by pre-surgery and current (time of survey completion). Logistic regression was used to identify predictors of score improvement for each of the questionnaires. Logistic models were only adjusted for ethnicity (white vs. others). Linear and bivariate analyses of age and type of surgery were not significant for any outcome measure, thus not included in adjusted models. All analyses were performed using Stata 17.1 (StataCorp, College Station, TX).

## Results

**Fig. 1:**
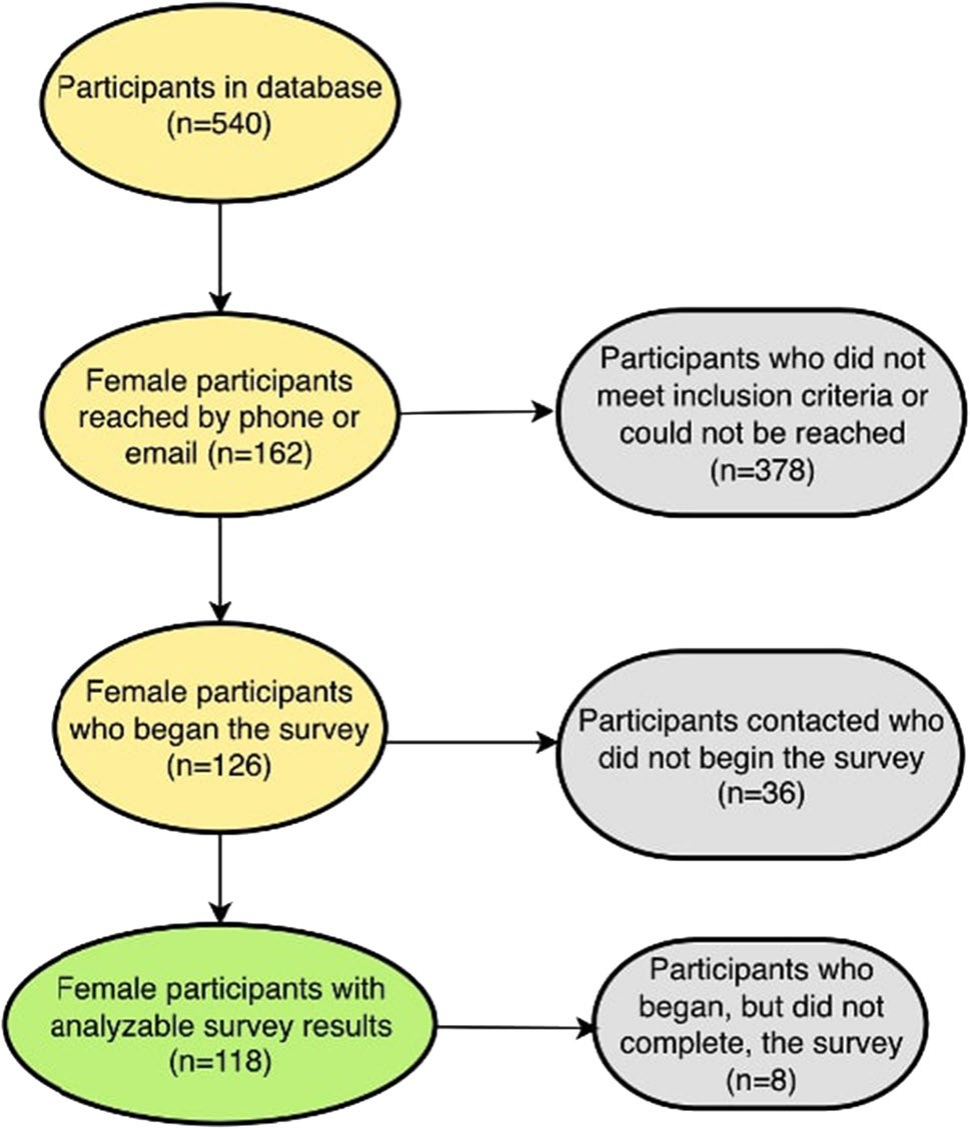
Participant recruitment flowchart

Of the 540 men and women who underwent MBS at UCSF during the study time period, 118 women met all criteria for inclusion in the study (Fig. 1). Participants were on average 50 years old (SD 13 year) at the time of survey administration, 44.6 ± 12.59 years old at the time of surgery, with a mean of 6.05 ± 6.04 years between surgery and survey administration. The majority of participants were white (54.2%), postmenopausal (50.8%), and had undergone sleeve gastrectomy (52.5%) (Table 1). All scores on all outcome measures were found to be normally distributed using histograms and verified using the Shapiro–Wilk test.

**Table 1:** Demographics

Variable	*N* = 118 unless listed	Total or mean ± SD^*^
*Current age*		50.41 ± 13.14
*Ethnicity†*		
*White*		64
*Hispanic/Latino*		29
*Black/African-American*		31
*Native America or American Indian*		7
*Asian/Pacific Island*		5
*Other*		5
*Height*	116	64.23 ± 3.03
*Weight before surgery*	116	294.99 ± 69
*Weight current*		203.81 ± 49.48
*BMI ‡ before surgery*	116	52.42 ± 12.64
*Current BMI*		36.27 ± 9.66
*% Total weight loss*	116	29.8% ± 13.1%
*% Excess BMI loss*	116	61% ± 25.6%
*Surgery type*	116	
*Lap band*		1
*Gastric bypass*		43
*Revisional*		10
*Sleeve gastrectomy*		62
*Menstrual status*		
*Premenopause*		41
*Perimeopause*		17
*Postmenopause*		60

^*^SD = *standard deviation*

^†^The total number of reported values for race/ethnicity is 141, reflecting individual participants ability to select more than one race/ethnicity when surveyed

^‡^*BMI* = *Body mass index*

### Urinary Function

The overall mean UDI-6 score amongst women was 24 ± 24 prior to MBS and 24 ± 26 at the time of survey administration (*p* = 0.458) (Table 2). Mean scores on all individual questions of the UDI-6 (frequency, urgency, incontinence with cough, drops, incomplete emptying, and dysuria) did not significantly change between the pre- and post-operative timepoints (Table 2). Logistic regression analysis showed factors associated with improvement of UDI-6 included pre-operative UDI score (OR = 1.05, *p* < 0.001) and post-operative improvement in POPDI scores (OR = 5.1, *p* = 0.002). No prior use of hormone therapy was associated with decreased odds of UDI improvement (OR = 0.3, *p* = 0.023) (Table 3).

**Table 2:** Overall inventory and individual subdomain change over time

*Outcome measure*	Prior (SD*)	*Current (SD)*	*p value*
*Mean Modified FSFI*^*†*^* Score*	15.65 (5.3)	14.21 (7)	**0.005**
*Desire*	2.95 (1.2)	2.99 (1.3)	0.067
*Arousal*	2.97 (1.6)	2.74 (1.9)	0.055
*Orgasm*	2.97 (1.6)	2.64 (1.9)	0.061
*Satisfaction*	3.14 (1.3)	3.08 (1.4)	**0.002**
*Pain*	3.64 (1.8)	2.76 (2.2)	**0.001**
*Mean UDI-6*^*‡*^* Score*	24.08 (24.1)	23.46 (26.5)	0.458
*Frequency*	1.11 (1.3)	1.15 (1.4)	0.337
*Urgency*	1.08 (1.4)	1.06 (1.4)	0.639
*UI coughing*	1.33 (1.5)	1.15 (1.4)	0.259
*“Drops”*	1.21 (1.4)	1.13 (1.4)	0.689
*Emptying*	0.58 (1.11)	0.73 (1.2)	0.505
*Pain*	0.46 (1)	0.41 (1)	0.831
*Mean POPDI-6*^*§*^* Score*	13.14 (14.8)	8.89 (13.8)	0.056
*Pressure*	0.71 (1.1)	0.39 (.9)	**0.047**
*Heaviness*	0.68 (1.1)	0.34 (.9)	**0.009**
*Bulge*	0.25 (.8)	0.25 (.7)	0.753
*Pushing*	0.55 (1)	0.42 (1)	0.633
*Incomplete*	0.85 (1.2)	0.64 (1.1)	0.384
*Bulge push*	0.11 (.5)	0.09 (.4)	0.880

All bold data is statistically significant (*p* < .05)

^*^Standard deviation

^†^Female Sexual Function Index

^‡^Urinary Distress Index

^§^Pelvic Organ Prolapse Distress Inventory

**Table 3:** Logistic regression model results for post- op improvement on the modified FSFI-6, UDI-6, and POPDI-6

	Adjusted model			Unadjusted model		
FSFI* parameters	Odds ratio	95% CI^†^ (lower, upper)	*p* value	Odds ratio	95% CI (lower, upper)	*p* value
*Post vs pre menopause*	0.189	0.060, 0.596	**0.004**	0.191	0.061, 0.602	**0.009**
*BMI*^*‡*^* change*	1.090	1.006, 1.182	**0.035**	1.091	1.008, 1.18	**0.031**
*White vs Non-white ethnicity*	2.162	0.829, 5.642	0.115			
*Prior hormone use vs. none*	0.671	0.251, 1.796	0.427	1.121	.0456, 3.616	0.510
*Prior FSFI score*	1.133	1.027, 1.249	**0.012**	1.127	1.024, 1.241	**0.015**
*BMI before surgery*	1.020	0.970, 1.071	0.446	1.021	0.971, 1.073	0.418
*POPDI-6*^*§*^* score improvement*	0.636	0.208, 1.940	0.426	0.625	0.209, 1.87	0.401
*UDI-6*^*¶*^* score improvement*	1.758	0.578, 5.348	0.320	1.527	0.611, 4.212	0.472
*POPDI-6 parameters*						
*Post vs pre menopause*	0.912	0.339, 2.457	0.856	1.039	0.401, 2.692	0.937
*BMI change*	0.944	0.882, 1.011	0.100	0.943	0.882, 1.009	0.091
*White vs Non-white ethnicity*	1.074	0.427, 2.703	0.879			
*Prior hormone use vs. none*	1.156	0.466, 2.872	0.755	1.165	0.348, 2.911	0.738
*BMI before surgery*	0.930	0.878, 0.985	**0.014**	0.930	0.878, 0.985	**0.013**
*FSFI score improvement*	1.448	0.496, 4.227	0.498	1.212	0.324, 2.057	0.560
*UDI-6 score improvement*	0.135	0.053, 0.342	** < .001**	0.157	0.064, 0.541	** < .001**
*UDI-6 parameters*						
*Post vs pre menopause*	0.564	0.192, 1.652	0.296	0.567	0.202, 1.745	0.453
*BMI change*	1.031	0.963, 1.104	0.385	1.039	0.975, 1.108	0.232
*White vs Non-white ethnicity*	2.636	0.948, 7.331	0.063			
*Prior hormone use vs. none*	0.3	0.106, 0.849	**0.023**	0.313	0.096, 0.852	**0.035**
*BMI before surgery*	1.018	0.962, 1.078	0.537	1.026	0.0972, 1.082	0.357
*POPDI-6 score improvement*	5.086	1.806, 14.317	**0.002**	5.35	1.938, 14.77	**.001**
*UDI-6 score improvement*	1.05	1.024, 1.076	** < .001**	1.049	1.025, 1.074	** < .001**
*FSFI score improvement*	2.215	0.701, 7.005	0.176	2.225	0.744, 6.959	0.228

All bold data is statistically significant (*p* < .05)

^*^FSFI = Female Sexual Function Index

^†^CI = Confidence interval

^‡^MI = Body mass index

^§^POPDI = Pelvic Organ Prolapse Distress Inventory

^¶^UDI = Urinary Distress Inventory

### Sexual Function

The overall mean modified FSFI scores amongst women was 15 ± 5 prior to surgery and 14 ± 7 at the time of survey administration (*p* = 0.005) (Table 2). Of the individual domains surveyed, only pain with sex showed statistically significant changes over time: decline with a mean pre-op score of 3.6 ± 1.8 and mean current score of 2.8 ± 2.2 (Table 2). Multivariate logistic regression demonstrated that post-menopausal respondents had decreased odds (OR = 0.2, *p* = 0.004) of FSFI improvement compared to pre-menopausal women, while increased BMI change (OR 1.1, *p* = 0.04) and lower pre-op FSFI score (OR 1.13, *p* = 0.012) increased odds of improvement (Table 3).

### Pelvic Organ Prolapse

The overall mean POPDI-6 score amongst women was 13 ± 15 prior to surgery and 9 ± 14 at the time of survey administration (*p* = 0.056) (Table 2). Of the six symptoms addressed by the POPDI-6, only pressure and heaviness showed statistically significant changes over time. Pressure showed an improvement from pre-op mean of 0.71 ± 1.1 to 0.39 ± 0.9 at time of survey (*p* = 0.047). Heaviness also improved from a pre-op mean of 0.68 ± 1.1 to 0.34 ± 0.9 current (*p* = 0.009) (Table 2). Multivariate logistic regression analysis revealed that UDI-6 score improvement (OR = 0.14, *p* < 0.001) and higher pre-op BMI (OR = 0.9, *p* = 0.014) decreased the odds of post-operative POPDI improvement (Table 3).

## Discussion

We report the first and largest cohort study to examine the experiences of women following weight loss surgery regarding prolapse, urinary, and sexual function outcomes. We found a high rate of clinically significant dysfunction across all three measured domains. Sexual health outcomes demonstrated either no significant change over time or decline post-operatively. All measured urinary health domains showed no significant change over time. For POP, only the domains of pressure and heaviness showed significant change over time, with both showing sustained improvement across timepoints. While patients did experience modest weight regain on average post-operatively, sustained BMI improvement was only associated with mildly improved sexual health outcomes. Hence, even the profound weight loss achieved with bariatric surgery failed to improve the self-reported prolapse, urinary, and sexual function of women.

An unexpected finding was that at baseline, women undergoing weight loss surgery reported a high degree of clinically significant symptoms of urinary incontinence, POP, and sexual dysfunction. Baseline UDI-6 scores in our study population were worse than those found in other populations known to be affected by significant urinary bother, such as pregnant multiparous women, patients with mixed urinary incontinence, and women with overactive bladder[10–13]. With regard to POP, POPDI-6 scores in our population demonstrated less bother (on average) than seen in patient cohorts either formally diagnosed with POP or whose POP symptoms met validated score cutoffs for POP[14, 15]. Sexual function in this population, as evaluated by modified FSFI-6 scores, was substantially lower than the validated cutoff score for clinically relevant sexual dysfunction, indicating significant dysfunction even when accounting for the missing FSFI domains[16, 17]. In summary, women with obesity who sought and received weight loss surgery on average had substantial pre-operative bother across all measured domains, often worse than in comparison to other high-risk populations.

This study has several limitations. We did not survey patients preoperatively, and so the design of this study was predisposed to significant recall bias when asking participants to remember facts and events, such as weight or sexual satisfaction, from the past. The effect of this recall was unpredictable, particularly when comparing small numeric changes in patient reported outcomes. The trend of improvement or decline over the intervening years since surgery, however, was likely preserved in the patient experience. Furthermore, survey data was limited by participant response. Given the sensitive nature of the topics addressed in our survey, it is possible that our results skewed towards those that experienced improvements in weight, sexual health, urinary health, or all three. Another inherent limitation was our use of an incomplete FSFI. Given the length of the original FSFI (19 questions), it was infeasible to have women complete the entire inventory at two time points (38 questions) on top of the UDI-6 and POPDI-6, which was why we excluded some of the FSFI questions and cannot report on the measure in its entirety. In addition, there is also the possibility that women sought treatment for bothersome sexual or urinary issues after weight loss surgery that our survey did not capture. While we captured hormonal status a surrogate measure of any treatment of peri/post-menopausal sexual dysfunction, this was far from comprehensive.

## Conclusion

In summary, we found that women who underwent MBS reported clinically significant levels of bother across urinary and sexual domains before surgery and also after significant weight loss. These data add to the growing body of evidence demonstrating the prevalence of these clinically relevant urinary and sexual symptoms in populations with obesity. Although MBS achieved excellent weight loss, most patients did not achieve a normal BMI even at their lowest weight, and such dramatic weight loss failed to meaningfully improve sexual and urological function. As such, it is important for those providing bariatric care and MBS to have conversations about sexual and urinary function with their patients and refer to urologists and other relevant specialists if symptoms are present, as symptoms are unlikely to resolve with MBS. Additionally, these findings can help guide counseling when discussing the possibility or anticipation of urinary and sexual dysfunction improvement after weight loss surgery.

## Supplementary Information

Below is the link to the electronic supplementary material.

Supplementary file1 (DOCX 35 KB)

## Data Availability

The data that support the findings of this study are available on request from the corresponding author, Dr. Lindsay Hampson. The data are not publicly available due to restrictions e.g. they containing information that could compromise the privacy of research participants.
